# Dissecting the Genetic Basis of Variation in *Drosophila* Sleep Using a Multiparental QTL Mapping Resource

**DOI:** 10.3390/genes11030294

**Published:** 2020-03-11

**Authors:** Brittny R. Smith, Stuart J. Macdonald

**Affiliations:** 1 Department of Molecular Biosciences, 4043 Haworth Hall, 1200 Sunnyside Avenue, University of Kansas, Lawrence, KS 66045, USA; 2 Center for Computational Biology, University of Kansas, Lawrence, KS 66047, USA

**Keywords:** sleep, activity, *Drosophila*, DSPR, MPP, QTL, expression QTL

## Abstract

There is considerable variation in sleep duration, timing and quality in human populations, and sleep dysregulation has been implicated as a risk factor for a range of health problems. Human sleep traits are known to be regulated by genetic factors, but also by an array of environmental and social factors. These uncontrolled, non-genetic effects complicate powerful identification of the loci contributing to sleep directly in humans. The model system, *Drosophila melanogaster*, exhibits a behavior that shows the hallmarks of mammalian sleep, and here we use a multitiered approach, encompassing high-resolution QTL mapping, expression QTL data, and functional validation with RNAi to investigate the genetic basis of sleep under highly controlled environmental conditions. We measured a battery of sleep phenotypes in >750 genotypes derived from a multiparental mapping panel and identified several, modest-effect QTL contributing to natural variation for sleep. Merging sleep QTL data with a large head transcriptome eQTL mapping dataset from the same population allowed us to refine the list of plausible candidate causative sleep loci. This set includes genes with previously characterized effects on sleep and circadian rhythms, in addition to novel candidates. Finally, we employed adult, nervous system-specific RNAi on the *Dopa decarboxylase*, *dyschronic*, and *timeless* genes, finding significant effects on sleep phenotypes for all three. The genes we resolve are strong candidates to harbor causative, regulatory variation contributing to sleep.

## 1. Introduction

Sleep is a phenomenon that has been recognized in a variety of vertebrate and invertebrate systems [1,2]. Although there is considerable variation in how sleep is presented across taxa [1,3,4], at a fundamental level, sleep is characterized by a state of behavioral quiescence. Within a species, natural variation among individuals in the duration and quality of sleep is extensive, with evidence available from humans, as well as from model systems such as mice [5] and flies [6,7].

Large cohorts of individuals assembled for human Genomewide Association Studies (GWAS) report significant phenotypic variation in typical sleep duration, timing, and quality [8,9,10,11]. Since a fraction of this variation is genetic in origin—for instance, the Single Nucleotide Polymorphism or SNP-based heritability of sleep duration has been estimated at 9.8% [8]—and because modern human GWAS employ hundreds of thousands of individuals, human geneticists have successfully identified, and in many cases replicated small-effect loci contributing to variation in sleep parameters in human populations. Such studies, conducted directly in humans, have implicated a number of biological pathways in the control of sleep variation, and have made connections between disease risk and sleep traits (e.g., [12]). Nonetheless, large-scale human GWAS typically lack phenotype replication (each individual genotype is phenotyped once), often use poorly-estimated phenotypes (for instance, self-reported measures of sleep duration), and must statistically combat the heterogeneity inherent in any study of humans (age, sex, diet, population structure, disease state, and so on). As a consequence, human GWAS results can be difficult to interpret. Compounding this issue is the inability to directly evaluate the functional consequences of human GWAS-identified genes in the whole organism, making it difficult to establish the mechanisms by which they impact variation in phenotype.

Extensive work in the *Drosophila melanogaster* model system, stemming from foundational studies by Hendricks et al. [13] and Shaw et al. [14] has demonstrated that flies rhythmically enter a state of inactivity that fulfills criteria for sleep. Five minutes of inactivity—the practical definition of sleep in most *D. melanogaster* studies—results in decreased behavioral responsiveness, a period of mechanical sleep deprivation results in sleep rebound, and stimulants like caffeine result in the expected decrease in sleep. The ability to directly examine sleep in a highly tractable model genetic system has led to significant insight into the neurobiology and regulation of sleep (reviewed thoroughly by [15]), and numerous genes and pathways with effects on sleep timing and during have been characterized in the system [16,17,18,19,20].

Many studies of sleep in flies leverage mutations identified in forward genetic screens (e.g., [16]), but it is also feasible to dissect naturally-occurring genetic variation contributing to fly sleep using sets of wildtype genotypes [6,21,22]. Such studies are complementary since genes that yield large-effect, loss-of-function mutant alleles may not necessarily be those that also contribute to naturally-segregating allelic variation. Here, we employ genotypes derived from the *Drosophila* Synthetic Population Resource, DSPR [23,24], a multiparental, advanced intercross panel of Recombinant Inbred Lines (RILs), and identify several Quantitative Trait Loci (QTL) contributing to various sleep phenotypes. We make use of extant, head-specific expression QTL (eQTL) data from the same genotypes [25] to examine regulatory correlates of sleep phenotypes, and find genes within mapped sleep QTL intervals that additionally possess *cis*-eQTL, and may be most likely to be functionally relevant sleep genes. Finally, using RNAi, we provide independent evidence that the genes *Dopa decarboxylase* (*Ddc*), *dyschronic* (*dysc*), and *timeless* (*tim*), all previously recognized as mediators of circadian rhythm or sleep, impact sleep phenotypes.

## 2. Materials and Methods 

### 2.1. Mapping Population

We employed genotypes derived from the DSPR for QTL mapping. To construct the DSPR, two sets of eight highly inbred strains were used to found a pair of synthetic recombinant populations (pA and pB). Each population was maintained as an independent pair of subpopulations (pA1 and pA2, pB1 and pB2) at large census size for 50 generations. The pairs of subpopulations therefore start with the same founders, but due to selection/drift during population maintenance may differ, locus-by-locus in their founder composition [24]. Subsequently, Recombinant Inbred Lines (RILs) were derived from each advanced generation intercross population via sibling mating. All 15 founder strains have been sequenced (the pA and pB populations share a single founder line), and all RILs were subjected to genotyping by sequencing to resolve the mosaic founder structure of each genome see [24] for additional details.

Rather than use the inbred RILs directly, for this study we elected to assay the F_1_ progeny of RIL-by-RIL crosses, which we refer to as DSPRF1 genotypes. This mapping design marginally reduces mapping power [23] but does minimize the potential for mapping QTL due to inbreeding, a potential concern for an activity-based behavioral trait like sleep. Each experimental heterozygous DSPRF1 genotype was generated by crossing 5 virgin females from a pA RIL to 5 males from a pB RIL. Each RIL was used just once, and crosses preserved the subpopulation structure of the DSPR by only crossing pA1 with pB2 RILs (referred to as “subpopulation 1”), and pA2 with pB1 RILs (“subpopulation 2”). We generated 787 unique test genotypes over 6 batches, arbitrarily assigning genotypes to batch based on numeric RIL identifiers, which have no relationship to genotype. In most cases, 8 experimental females of a given genotype were taken from a single cross vial from a single batch for phenotyping. However, a small subset of genotypes was generated independently across batches.

### 2.2. Phenotyping Assay

Cross vials were visually inspected twice a day, and adults were discarded after 1–2 d to maintain approximately equivalent egg densities across vials. Experimental virgin female DSPRF1 flies were collected over CO_2_ anesthesia and housed in single-sex groups of 8–12 animals for 3–4 d prior to the assay. Maintaining a similar density of flies per vial prior to the assay should minimize the effects of variable crowding on sleep architecture [26]. For the assay, 3–4 d old experimental DSPRF1 virgin female flies were aspirated singly and without anesthesia into polycarbonate activity monitor tubes (5 mm diameter × 65 mm length; TriKinetics, Inc.) containing a small quantity of media (Nutri-Fly Molasses Formulation, Genesee Scientific). The tubes were then plugged, and inserted into DAM2 *Drosophila* Activity Monitors (TriKinetics, Inc.). Activity levels—measured as the number of times a fly breaks an infrared beam running across the center of the monitor tube—were automatically recorded by the DAMSystem3 data collection software (TriKinetics, Inc.). We recorded activity every minute for every fly starting at 08:00 (lights on) the day after loading into monitors and continuing for a total of four 24 h periods. Thus, during the assay, test flies were between 3–4 and 8–9 d old. All strains and experimental genotypes were reared, crossed, and assayed at 25 °C, 50% relative humidity, using a 12 h:12 h light: dark cycle with lights on at 08:00.

Subsequently, we measured several activity- and sleep-related traits for every experimental female using a custom script written for the R programming language [27]. In common with previous work [14], we define sleep as a period of 5 min of inactivity. In addition to the length of time spent sleeping during any given 12 h light or dark period, we also count the number of sleep bouts (continuous periods of inactivity), the length of these sleep bouts, and the total activity while awake. For each genotype, these values are averaged over replicate animals, and over the multiple days of the experiment.

### 2.3. QTL Mapping in the DSPR

We calculated a mean phenotype for each of 787 DSPRF1 genotypes to use for mapping (Supplementary Table S1). In the case of sleep bout time in the dark, and particularly in the light, there is clear heteroscedasticity in the distributions, with the variance among animals of a genotype markedly increasing with genotype mean. For these phenotypes, we carried out QTL mapping on genotype means following a log_10_ transform.

Separately for each trait, we regressed mean phenotype on the 16 additive probabilities that correspond to the probabilities the maternal RIL is derived from each of the eight pA founders, and the probabilities the paternal RIL is derived from each of the eight pB founders. We additionally included a covariate to control for differences in phenotype due to subpopulation. Genomewide significance thresholds for QTL detection were found through 1000 permutations of the data [28], and confidence intervals on QTL locations were taken as 3-LOD drops from the LOD peaks [23]. Mapping was implemented using the DSPRqtl R package [29,30], and additional details on the approach and analytical model have been presented previously [23,24,25].

### 2.4. Correlations Among Strain Effects at QTL

Given the multiparental nature of the DSPR population, at mapped QTL we can estimate the mean phenotype associated with each founder haplotype. For a pair of QTL that co-localize, a natural question is whether there is evidence that the founder effects are similar (if so, this might imply common genetic control of trait variation). We use Pearson correlation coefficients among founder haplotype means estimated at each QTL to address this, only employing founder means derived from at least 10 observations.

### 2.5. Functional Testing via RNAi

We functionally tested three plausible candidate genes—*Dopa decarboxylase* (*Ddc*), *dyschronic* (*dysc*), and *timeless* (*tim*)—for effects on sleep using RNAi knockdown via the Gal4-UAS system. Two Gal4 sources were employed: UAS-*Dicer2*; +; *elav*-Gal4/TM6C, *Sb*^1^, *Tb*^1^ (a gift from Troy Zars, University of Missouri) to drive Gal4 pan-neuronally, and *y*^1^ w*; *elav*-GeneSwitch-Gal4 (Bloomington *Drosophila* Stock Center, BDSC stock number 43642) to drive Gal4 in adults only after feeding animals RU486 (mifepristone). To generate each target genotype, we crossed 10 virgin females from the UAS or control strain to 5 males from the Gal4 strain, establishing 7–10 replicate cross vials, and collected experimental virgin females in groups of 8–12. Experimental Gal4-UAS animals harboring *elav*-Gal4 were treated and assayed as described above for the DSPRF1 animals. Animals carrying *elav*-GeneSwitch-Gal4 were maintained on regular food for one day following collection, were transferred onto media supplemented with 100 μg/mL RU486 for two days, then were aspirated singly into monitor tubes holding caffeine test media supplemented with 100 μg/mL RU486. The activity-based assay was then carried out as described above for the DSPRF1 animals.

Transgenic RNAi Project, TRiP [31] UAS-RNAi and co-isogenic control strains were obtained from the BDSC, specifically stock numbers 35788 (UAS-*Luciferase* control), 27030 and 51462 (UAS-*Ddc-RNAi*), and 29583 and 40864 (UAS-*tim-RNAi*). Vienna *Drosophila* Resource Center (VDRC) [32] UAS-RNAi and controls were also used: strains from the VDRC-GD library, which each have a *P*-element-derived transgene, specifically stock numbers 60000 (*w^1118^* control strain), and 14082 and 28945 (UAS-*dysc-RNAi*), and strains from the VDRC-KK library, which have phiC31-derived transgenes, specifically 60100 (landing site control strain), 109881 (UAS-*Ddc-RNAi*) and 11019 (UAS-*dysc-RNAi*).

### 2.6. Data and Scripts

Much of the processed data generated for the study is available in the supplementary tables. All raw data, all processed data, and all R scripts, including those used for sleep/activity phenotype measurement, are freely available as a Dryad repository (https://doi.org/10.5061/dryad.6wwpzgmv8).

## 3. Results and Discussion

### 3.1. Extensive Genetic Variation for Sleep and Activity Traits in the DSPR

We measured a suite of phenotypes in 787 F_1_ heterozygous genotypes derived from the DSPR [23], using an average of 8.7 virgin females per DSPRF1 genotype, and averaging phenotype over four sequential 24 h periods of data collection while flies were 3–9 d old. Fifty-one DSPRF1 genotypes were independently generated and assayed across two experimental batches, and the correlation between the batch-specific phenotype means is high (e.g., light-phase sleep time, Pearson′s *r* = 0.72, *p* < 10^−8^; Supplementary Figure S1) giving us confidence in the accuracy of our phenotypic measures, and their suitability for mapping.

**Figure 1:**
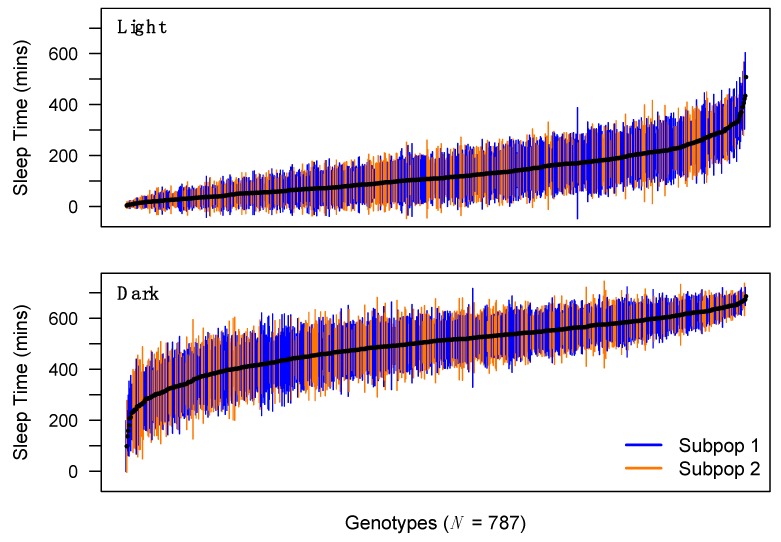
Distribution of sleep time across DSPRF1 genotypes. Mean sleep time (+/− 1SD) during the light and dark period for each of 787 trans-heterozygous virgin female DSPRF1 genotypes. Genotypes derived from subpopulation 1 (progeny of crosses between pA1 × pB2 Recombinant Inbred Lines (RILs)) and subpopulation 2 (progeny of crosses between pA2 × pB1 RILs) are highlighted in different colors. Since the subpopulations differ slightly in their founder composition, they can exhibit average differences in phenotype. For instance, the subpopulations differ in light-phase sleep time (Welch’s *t*-test, *P* < 0.001), but not in dark-phase sleep time (*P* = 0.97). We corrected for subpopulation-to-subpopulation effects during QTL mapping for all sleep/activity phenotypes.

We see considerable among-genotype variation in phenotype for all traits measured in the DSPRF1 (Figure 1; Supplementary Figure S2; Supplementary Table S1). Genotype means range from 98.3 to 686.1 min of dark-phase sleep and 3.8 to 507.8 min of light-phase sleep per 12 h period (i.e., 720 min). A previous study assayed sleep in virgin flies from 168 inbred strains of the *Drosophila* Genetic Reference Panel, DGRP [6], and found a fairly similar range of dark-phase sleep in females (121.9–688.7 min), but a distinct range of light-phase sleep values (94.1–639.8 min). There was greater sleep during the light period in the inbred DGRP flies compared with outbred DSPRF1 flies (Supplementary Figure S2). Indeed, 42% (97%) of DSPRF1 light-phase sleep time genotype means are below the minimum (average) DGRP strain mean. This difference in overall light-phase sleep time distribution could be due to differences in the details of the assay (e.g., media formulation), differences in the allelic variation contained within the mapping panels [23,33,34,35], differences in zygosity, or a combination of these factors.

To examine whether light-phase sleep in virgin female flies is increased in inbred, homozygous lines relative to heterozygous genotypes, perhaps as a result of reduced activity due to inbreeding depression, we compared sets of DSPR RILs and DGRP inbred lines to their cross progeny. We see no clear evidence for a difference in light- or dark-phase sleep between homo- and heterozygous animals (Supplementary Figure S3). However, when we compared our measures of light- and dark-phase sleep for 24 inbred DGRP lines with those reported in Harbison et al. [6], while the correlations are high (*r* = 0.72 for light-phase sleep time and *r* = 0.74 for dark-phase sleep time, *p* < 0.0001), the actual measures of fly sleep are much lower in our study. The average light- and dark-phase sleep for this subset of lines from the Harbison et al. [6] study is 377 and 558 min, respectively, while our measures are 223 and 404 min, respectively. It appears likely that one or more differences in environment and assay conditions are responsible for much of the difference in average light-phase sleep time between the Harbison et al. [6] DGRP study and the DSPRF1 data presented here.

We generated estimates of broad-sense heritability for sleep phenotypes in the DSPRF1 by estimating variance components from a linear mixed model using the nlme R package [36], nesting genotype within subpopulation (see also [37]). Heritability for all traits in our mapping population is substantial: light-phase sleep time (51.4%), dark-phase sleep time (53.5%), light waking activity (46.3%), dark waking activity (36.0%), light bout number (47.5%), dark bout number (38.2%), light bout length (25.0%), and dark bout length (37.1%). These heritability values are generally consistent with those obtained for the same traits in the DGRP inbred line GWAS panel [6] and demonstrate that considerable segregating variation for sleep and activity parameters exists within *Drosophila* populations. Indeed, recent work by Harbison et al. [22] has leveraged this genetic variation to create long- and short-sleeping *Drosophila* lines via artificial selection and saw a rapid divergence in phenotype over a modest number of generations of selection.

**Figure 2:**
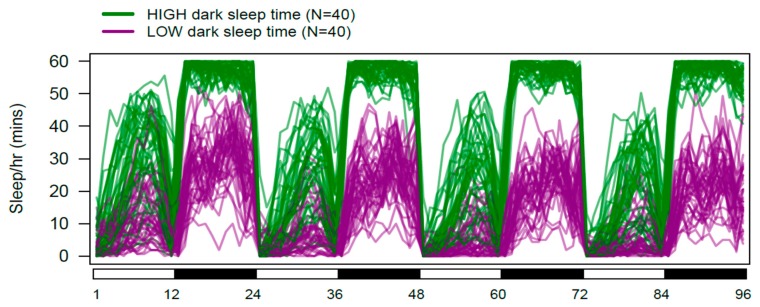
Daily sleep in DSPRF1 genotypes exhibiting extreme dark-phase sleep time. The 40 DSPRF1 genotypes with the highest, and the 40 with the lowest average dark-phase sleep time were identified. For each of these genotypes, we calculated the average sleep time per hour per fly throughout the 96 h data collection period. The figure shows 80 overlapping sleep profiles, with those in green from high dark-phase sleep genotypes, and those in purple from low dark-phase sleep genotypes.

Among-trait correlations in the DSPRF1 genotype panel are generally modest, but extremely significant given the size of the dataset (Supplementary Table S2). Given that sleep is measured as a function of activity level, it is unsurprising that sleep time is inversely correlated with activity level (light, Pearson’s *r* = −0.23, *p* < 10^−10^; dark, *r* = −0.15, *p* < 10^−4^). In the dark phase, the total amount of sleep is negatively associated with the number of sleep bouts and positively associated with bout length (bout number, *r* = −0.57, *p* < 10^−40^; bout length, *r* = 0.72, *p* < 10^−40^), implying that those genotypes sleeping for longer during the dark period do so using fewer, longer bouts of rest. The situation is different during the light period, where total sleep time is positively associated both with the number and duration of sleep bouts (bout number, *r* = 0.79, *p* < 10^−40^; bout length, *r* = 0.77, *p* < 10^−40^), implying those genotypes sleeping longer in the light period rest in more, longer bouts. Finally, sleep time during the light and dark periods is positively correlated (*r* = 0.43, *p* < 10^−40^; Supplementary Figure S4). Figure 2 highlights this result, showing the daily sleep patterns for the 40 DSPRF1 genotypes with the lowest and highest average levels of dark-phase sleep. It is clear that in addition to differing in the amount of rest obtained in the dark, these two groups of genotypes also differ with respect to the average amount of sleep during the light period. As a result of the correlations among traits, we expect some overlap in the mapped loci contributing to variation in the individual sleep and activity traits we assay.

### 3.2. Loci Contributing to Genetic Variation in Sleep

**Figure 3:**
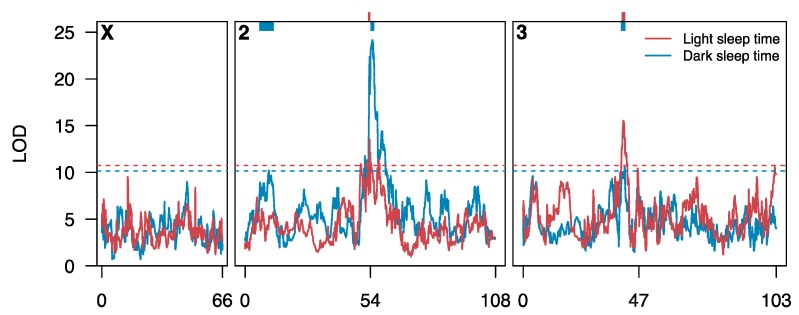
Quantitative Trait Loci (QTL) identified for sleep time in the DSPRF1. Each curve shows a genome scan for loci affecting sleep time in the light (red) or dark (blue). Genetic positions 54 and 47 on chromosomes 2 and 3, respectively, represent the positions of centromeres. The horizontal dashed lines represent genomewide 5% permutation thresholds (light: LOD = 10.7, dark: LOD = 10.1). The colored bars at the top of the plot represent the 3-LOD drop intervals of the five QTL mapped for sleep time.

**Figure 4:**
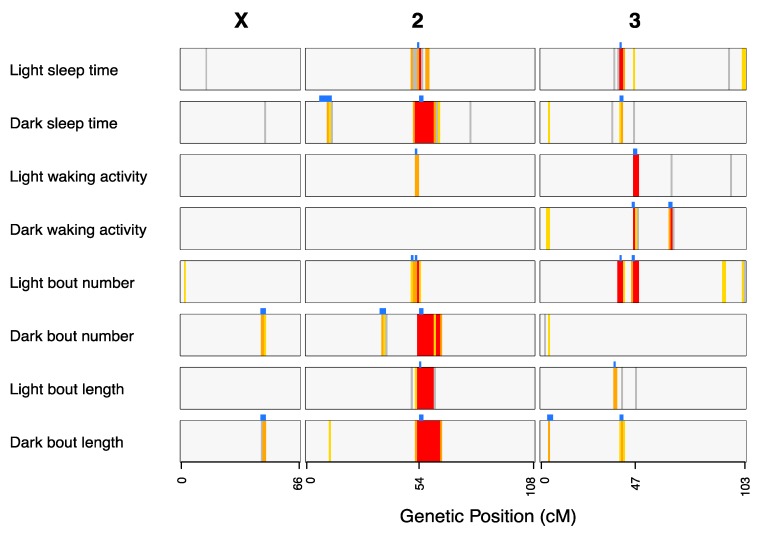
Locations of QTL mapped for all sleep and activity traits in the DSPRF1. The genomewide LOD scores for each trait are depicted as colored vertical stripes within each bar, with colors indicating whether the score survives a trait-specific permutation-based critical threshold (1% = red, 5% = orange, 10% = yellow, 20% = gray; Supplementary Table S5). The blue blocks above each phenotype bar highlight the 3-LOD drop intervals of mapped QTL that survive a 5% threshold.

We independently mapped QTL contributing to variation in each of eight traits, mapping 2–4 QTL per trait (Supplementary Table S3; Supplementary Table S4; Supplementary Table S5). Figure 3 provides the positions of those QTL mapped for light-phase sleep time (two QTL) and dark-phase sleep time (three QTL), and Figure 4 shows the positions of all QTL. We mapped a total of 22 trait-specific QTL (Supplementary Table S4), with each explaining on average 7.6% (5.5–13.9%) of the among-genotype variation for a trait. Making the assumption that QTL are independent, and act additively, we explain an average of 20.9% (13.3–32.3%) of the genetic variation for each trait with mapped QTL, capturing reasonably large fractions of the variation in relatively small segments of the genome.

The DSPRF1 mapping panel, as with any multiparental cross, segregates for multiple alleles at every chromosomal position. This provides the opportunity to estimate the effects on phenotype conferred by each founder-derived haplotype at QTL. Naively, we might anticipate that founder alleles at QTL fall into two groups (i.e., “high” and “low”), indicating a single, biallelic variant is responsible for the QTL. However, the founder effects for the 22 sleep/activity QTL do not exhibit this pattern (Supplementary Figure S5; Supplementary Table S6), and instead indicate that QTL frequently exhibit a pattern suggestive of the presence of allelic series. This is perhaps unsurprising because we and others have previously shown that mapped QTL and eQTL in multiparental populations show evidence for the segregation for >2 alleles [25,38], allelic series have been found at genes contributing to variation in other complex traits (e.g., [39]), allelic heterogeneity is evident in human GWAS [40], and Mendelian diseases are clearly multiallelic [41]. Nonetheless, given that our QTL encompass multiple genes (Supplementary Table S4), we cannot discount the possibility that QTL are generated by the action of variants at several closely-linked genes (i.e., QTL are multigenic rather than multiallelic). In addition, there is considerable founder genotype frequency variation in the DSPR, and at any given location the fraction of RILs harboring a given founder is often very different from the expected 1/8 [24]. This is likely due to drift and/or selection during maintenance of the synthetic populations from which the RILs were derived. As a result, the phenotype conferred by each founder haplotype at a position is not estimated over an entirely random set of genetic backgrounds, potentially leading to difficulty accurately estimating founder effects. To the extent that estimates are biased, our interpretation of non-biallelic QTL may be incorrect.

A particular strength of multiparental, advanced generation genetic mapping populations is they enable generally higher mapping resolution than is possible with standard, two-parental F_2_/backcross mapping [42,43]. We mapped QTL to intervals averaging 1.8 cM, from a minimum of 0.2 cM to a maximum of 6.3 cM. Ignoring those seven QTL mapping to intervals encompassing, or immediately adjacent to autosomal centromeres—sites of suppressed recombination—the physical size of mapped QTL averages 1.02 Mb and encompasses an average of 93 protein-coding genes (Supplementary Table S4).

### 3.3. QTL Overlap Among Traits

Given the correlations among phenotypes in the DSPRF1 mapping panel (Supplementary Table S2), we anticipated that some fraction of QTL would overlap among traits. Considering coincident QTL to be those whose 3-LOD drop support intervals overlap, we identified a total of 12 “QTL groups” (Table 1)—7 representing QTL found uniquely for a single trait, and 5 multiple QTL groups where trait-specific QTL overlap (Figure 4; Table 1). For 3/5 of these multiple QTL groups (QG1, QG5, QG7), correlations among founder strain effects (Supplementary Table S6) for all pairs of trait-specific QTL were significant (10^−8^ < *p* < 0.05), implying the site, or sites contributing to variation in each trait is the same, or at least are in strong linkage disequilibrium. For QG10, where QTL for four traits overlap (Table 1), strain effects are highly correlated for the light-phase sleep time and light bout number QTL (Pearson’s *r* = 0.98, *p* < 10^−8^), and strain effects are highly correlated for the dark-phase sleep time and dark bout length QTL (Pearson’s *r* = 0.97, *p* < 10^−8^), but none of the other pairs of QTL show correlated founder effects. For QG11, where trait-specific QTL for light waking activity, dark waking activity, and light bout number are coincident, none of the pairwise strain effect correlations are significant at the 20% level. The observation that overlapping QTL do not show correlated strain effects could imply the genetic bases of the trait-specific QTL are different, and QTL overlap stochastically, or that the same allelic variants have different effects depending on the trait. Only further fine mapping, or subsequent functional genetics testing would be able to distinguish these possibilities.

Four QTL groups (QG4, QG5, QG6, QG7) do not overlap, but are all close to each other around the chromosome 2 centromere (Figure 4). Recognizing that inbreeding of the DSPR genotypes was less efficient around centromeres [24], and that the physical size of peri-centromeric haplotype blocks is larger, while we consider these four QTL independent based on our mapping data, it is possible that they represent the same molecular variants. QTL residing at, or very near centromeres will always be incredibly challenging to dissect to the molecular level.

For those QTL significant for a just a single trait, it is clear that, in some cases, the same positions show high LOD scores for other traits, albeit with scores that do not survive a 5% genomewide threshold (Figure 4). For instance, QG8 for dark bout length (Table 1) at the tip of chromosome 3L is in the same position as peaks surviving a 10% genomewide threshold for dark-phase sleep time, dark waking activity, and dark bout number (Figure 4). Thus, many of the QTL we report as being associated with only a single trait may in reality be pleiotropic.

In order to reduce the size of the genomic windows implicated by our study, we make the assumption that the factor contributing to each member of a QTL group is the same. We recognize that this assumption may not always be valid, but it does enable prioritization of the smallest number of the most likely candidate causative genes for subsequent exploration. We define the size of each QTL group as the smallest interval encompassed by all overlapping trait-specific QTL (Table 1; Supplementary Table S7). The 12 QTL groups implicate 8.6% of the genetic map, and 15.0% of the physical genome, with the difference largely due to QG7 that resides over the chromosome 2 centromere (Table 1). We used FlyBase [44] to identify genes previously implicated in the genetic control of sleep and circadian rhythms by finding those genes tagged with the Gene Ontology (GO) terms “circadian rhythm” (GO:0007623), “regulation of circadian rhythm” (GO:0042752), and “sleep” (GO:0030431), along with those tagged with the FlyBase controlled vocabulary term “sleep defective” (FBcv:0000705). We identified 249 unique protein-coding genes with these searches (Supplementary Table S8), 24 of which resided within the 12 QTL groups (Table 1). With the exception of QG5 all QTL groups harbored at least one sleep/circadian rhythm candidate gene (Supplementary Table S7), such as the classic clock genes *timeless* (*tim*, QG2) and *cryptochrome* (*cry*, QG12).

### 3.4. Resolving Plausible Regulatory Genes via eQTL Mapping

Current evidence indicates that a fraction, probably a substantial fraction, of intraspecific variation for complex traits is due to regulatory variation that modulates the expression of causative genes [45,46,47]. For a behavioral trait such as sleep, one might anticipate that a regulatory factor active in the nervous system would be of principal interest. Thus, genes within QTL intervals identified for sleep-related traits, that additionally segregate for *cis*-eQTL in a behaviorally-relevant tissue, are strong candidates to causally contribute to phenotypic variation.

Previously, we carried out an array-based expression QTL (eQTL) study to identify loci contributing to regulatory variation in female *D. melanogaster* heads [25]. We used 596 DSPRF1 genotypes (567 of which are an exact subset of the 787 phenotyped in the current study) and identified 7922 eQTL for 7850 transcripts, including 7775 *cis*-eQTL. Expression of 1026/1390 protein-coding genes within our mapped sleep QTL groups were successfully interrogated by King et al. [25], and 552 of these showed a *cis*-eQTL (Table 1; Supplementary Table S9). Approximately one-fifth to one-half of the genes implicated by each QTL group harbor a *cis*-eQTL, and under the assumption QTL confer effects on phenotype via regulatory changes, such genes are excellent candidates to possess functional regulatory variation. Notably, of the 24 sleep/circadian rhythm genes present within QTL groups, 13 additionally possess local eQTL (Supplementary Table S7), including *tim* (QG2), *Dopa decarboxylase* (*Ddc*, QG6), a critical mediator of dopamine biogenesis, and *dyschronic* (*dysc*, QG10) and *circadian trip* (*ctrip*, QG11), both regulators of circadian rhythm.

If the variant(s) underlying both a *cis*-eQTL and a phenotype QTL are the same (i.e., the effect of the QTL on phenotype is due to local regulatory variation), we would expect that there should be a correlation between the founder strain effects at the overlapping *cis*-eQTL and QTL. We calculated pairwise correlations between the strain effects for each of the 22 trait-specific QTL (Supplementary Table S6) and each overlapping *cis*-eQTL (Supplementary Table S9). A total of 59 genes show a nominally significant (*p* < 0.05) founder strain effect correlation between the local eQTL and one or more trait-specific QTL (1–10 genes per QTL group; Supplementary Table S10). Of the 13 sleep/circadian rhythm genes with local eQTL that reside within QTL groups, three show significant eQTL/QTL strain effect correlations (*p* < 0.05); *Elongator complex protein 3* (*Elp3*, QG2), *taiman* (*tai*, QG3), and *Ecdysone receptor* (*EcR*, QG7).

We note that while some of the eQTL/QTL strain effect correlations are strong (for instance, 131/1828 have Pearson correlation coefficients of *r* > |0.6|), none survive Bonferroni correction for multiple tests. This is due, at least in part, to the limited number of founders for which we have effect estimates at QTL, which has a maximum number of 16 but in practice is fewer (mean = 10.7, range = 8–15) due to the marked variation in founder representation across the genome in the DSPR RILs [24]. Thus, the absence of a very strong eQTL-QTL founder strain effect correlation does not necessarily imply the target gene possessing the *cis*-eQTL is not a true regulator of the phenotype.

### 3.5. Correlations between Gene Expression and Phenotype

Since our phenotyping and expression profiling studies employed the same set of 567 genotypes, we were additionally able to correlate transcript expression level with phenotype score. We identified a total of 91 genes whose expression level correlated with at least one phenotype at a per-trait False Discovery Rate threshold of 5% (Supplementary Table S11). Although these correlations are highly significant, the correlation coefficients are modest, with a maximum Pearson *r* = |0.24|, presumably due both to estimation error, and because any relationship between the expression level of a causative gene and the phenotype it controls is not direct. Twenty-three of these correlated genes reside within our set of 12 QTL groups (Supplementary Table S7), although most (14) are present within QG7, which is by far the largest physical QTL interval. Sixteen of the 23 genes with expression correlations also exhibit local eQTL, but just one is a recognized sleep/circadian rhythm candidate gene, *EcR* (QG7). The expression of *EcR* is significantly correlated with dark-phase sleep time (*r* = −0.16, *p* < 0.0005), dark bout number (*r* = 0.18, *p* < 10^−4^), and dark bout time (*r* = −0.20, *p* < 10^−5^). Ishimoto and Kitamoto [48] demonstrated that ecdysone level impacts sleep time and sleep homeostasis, supporting the idea that *EcR* regulates sleep. However, in their study compound, heterozygous loss-of-function *EcR* mutants showed reduced sleep, whereas our correlation implies reduced sleep is associated with higher *EcR* expression.

### 3.6. Overlap with Loci Previously Implicated in the Genetic Control of Natural Variation in Sleep in Flies

Harbison et al. [6] used a GWAS design employing the DGRP [33,35] to measure the same sleep/activity traits we describe here using very similar experimental methods. That study identified 2427 unique associations across both sexes and five traits (dark-phase sleep time, total waking activity, light bout number, light bout length and dark bout length) at a False Discovery Rate of 1% (data taken from additional file nine of [6]). Most of the reported variants were associated with only one trait, while 3.6% (88/2427) showed associations for exactly two traits. Given the relatively low power of the DGRP design to identify modest-effect associations [49], it is reasonable to think that in reality more of the variants contribute to more than one trait. Notably, 82.4% of the total set of 2515 variant–phenotype associations were found for a single trait, light bout length.

One hundred and eighty-seven of the 2427 unique variants associated with phenotype by Harbison et al. [6] are present within our 12 QTL groups. Given that our QTL groups encompass 18.95 Mb (15%) of the *Drosophila* euchromatic genome, we might expect ~364 GWAS hits within QTL intervals purely by chance, with no functional overlap in sleep genetic architecture between the DGRP and DSPR. A simple resampling test—counting the number of associated variants within 12 randomly-positioned, non-overlapping regions of equivalent size to true mapped QTL groups—confirms that there is no enrichment of DGRP GWAS hits within DSPRF1 QTL; Over 1000 runs, the 95th percentile on the number of GWAS hits within simulated QTL was 523. (A simulation that excluded the very large physical interval implicated by QG7 revealed a similar lack of enrichment of GWAS associations within QTL.) We have previously seen a similar lack of overlap between mapping results in the DGRP and DSPR for three toxicity and stress tolerance traits [50,51,52]. This lack of replication is likely due to a combination of power deficits [49], differences in environmental and assay conditions (see above), and the fact that many variants do not segregate in both the DGRP and DSPR [34].

Nonetheless, the lack of enrichment does not exclude the possibility that those DGRP-derived associations that do reside within mapped DSPR QTL regions are causative. Of the 187 unique associated variants, 76 (40.6%) are in transcribed regions, and implicate 55 unique genes within QTL groups (Supplementary Table S7). Twenty-nine of these genes additionally possess local eQTL, although just one—*tim*—is also a well-known sleep/circadian rhythm gene.

### 3.7. Functional Testing of Candidate Sleep Genes via Neuron-Specific RNAi

Eight QTL groups—QG2, 3, 6, 7, 8, 10, 11 and 12—harbor one or more genes that have recognized roles in sleep/circadian rhythm control and segregate for a *cis*-eQTL. To attempt to validate the effects of a subset of these genes via RNAi, we selected one from each of the three physically-smallest QTL groups that contribute to sleep time (Table 1): *tim* which is present under QG2 (QTL for dark-phase sleep time), *Ddc* which is present under QG6 (QTL for light-phase sleep time), and *dysc* which is present under QG10 (QTL for light- and dark-phase sleep time, light bout number, and dark bout length). We tested RNAi knockdown of each gene via the Gal4-UAS system using 2–3 different UAS transgenes per target gene, 61–147 experimental individuals per genotype, and testing both pan-neuronal knockdown via an *elav*-Gal4 driver, and—for a subset of the UAS transgenes—adult-specific neuronal knockdown via an RU486-inducible *elav*-GeneSwitch-Gal4 driver (see Materials and Methods). The adult-specific knockdowns were carried out in an effort to avoid any developmental effects of the RNAi. Supplementary Table S12 presents a statistical analysis of differences between each pair of matched control and experimental RNAi genotypes for each phenotype scored.

**Figure 5:**
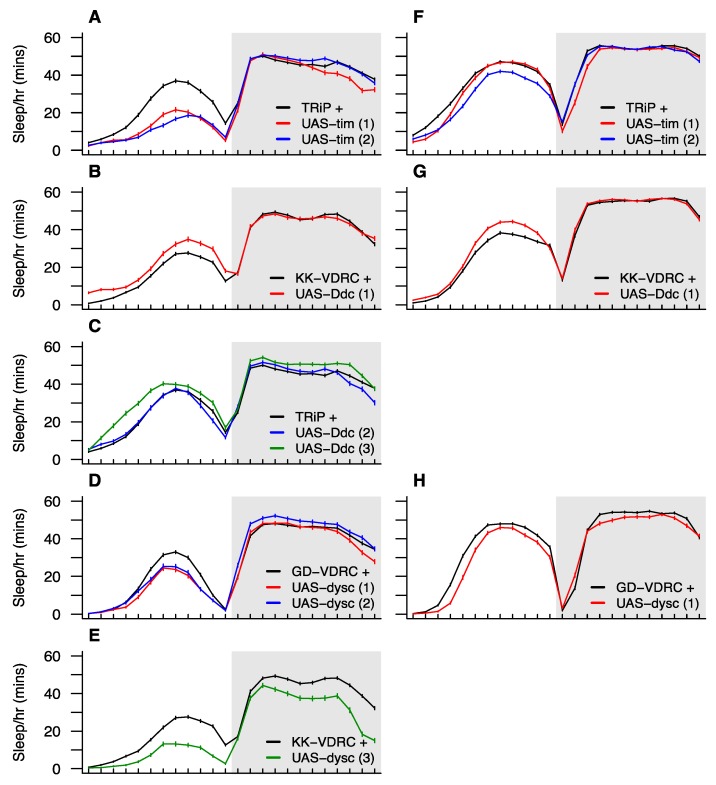
Sleep time profiles for RNAi knockdown of candidate genes. We tested three candidates, each residing under a different QTL group: *timeless* (QG2), *Dopa decarboxylase* (QG6), and *dyschronic* (QG10). For each gene, we tested multiple UAS-RNAi transgenes (shown in red, blue, and green lines) against the appropriate control strain (shown in black). Panels **A**–**E** show results of RNAi driven using the pan-neuronal *elav*-Gal4 driver, and panels **F**–**H** show the results of RNAi for a subset of the strains driven by *elav*-GeneSwitch-Gal4 specifically in adult neurons using the RU486-inducible system. In the figure, simple codes are provided to describe transgenes and controls. Stock numbers for these are: TRiP + (BDSC 35788), UAS-tim #1 and #2 (29583 and 40864), UAS-Ddc #2 and #3 (27030 and 51462), KK-VDRC + (VDRC 60100), UAS-Ddc #1 (109881), UAS-dysc #3 (11019), GD-VDRC + (60000), and UAS-dysc #1 and #2 (14082 and 28945). More detail is provided in Supplementary Table S12.

The *timeless* gene is a classic clock gene [53,54] that is critical to the regulation of circadian rhythms. Knockdown of *tim* in all neurons throughout life leads to a significant reduction in light-phase sleep time (Figure 5A), which is associated with significantly fewer and shorter sleep bouts. We also observed significantly reduced light-phase sleep time following RNAi of *tim* in adult neurons only, although the effect is pronounced only in one of the two UAS transgenes (blue curve, Figure 5F). This effect appears to be due to a reduced number of sleep bouts during the light period (Supplementary Table S12). Despite QG2 contributing to dark-phase sleep time (Table 1), *tim* RNAi did not routinely impact this phenotype, potentially suggesting that variation at *tim* is not the driver of the QTL. Nonetheless, *tim* is an excellent a priori candidate to impact sleep-related traits, and RNAi knockdown is not necessarily expected to phenocopy the effects of any causative variation segregating at the gene. Additionally, the QG2 region harboring *tim* also encompasses a subthreshold LOD peak for sleep bout length in the dark (Figure 4), and adult neuron-specific *tim* knockdown markedly reduces this phenotype (Supplementary Table S12), providing some—admittedly circumstantial—evidence that *tim* has pleiotropic effects on more than one sleep phenotype.

The protein product of the *Dopa decarboxylase* gene is involved in the production of dopamine and serotonin [55], and dopamine signaling has been broadly implicated in regulating sleep/wake cycles (see [15]). Naturally-segregating variation at *Ddc* has been implicated in locomotor activity [56], a phenotype that is highly relevant for sleep in flies, which is inherently an activity-based measure. Additionally, Svetec et al. [7] identified circadian time-dependent differential expression at *Ddc* between populations with a significant difference in sleep. Of the three UAS transgenes we employed for *Ddc* RNAi one failed to lead to any significant change in phenotype (blue curve, Figure 5C). One showed a significant increase in light-phase sleep time with both Gal4 drivers (Figure 5B,G) that is associated with a greater number of bouts of sleep and a reduced level of activity while awake during the light period (Supplementary Table S12). The third transgene showed the same pattern in the light period following RNAi knockdown of all neurons throughout life, and additionally showed an increase in sleep during the dark period (green curve, Figure 5C), that appears to be accomplished through fewer, longer bouts of sleep (Supplementary Table S12). QG6, under which *Ddc* resides, influences light-phase sleep time (Table 1). However, the region is at the chromosome 2 centromere where LOD scores for many traits are above threshold. Thus, it is plausible that segregating variation at *Ddc* does indeed affect multiple traits as these RNAi data might suggest. A potential concern with these *Ddc* RNAi results is that activity while awake is reduced and light-phase sleep time is increased. Arguably, these are the responses one might expect from an unhealthy, inactive genotype, which might imply that reducing *Ddc* neuronal expression has broad negative effects on health and is not specific to sleep. 

Loss of function of the *dyschronic* gene results in arrhythmic locomotion [57]. All four UAS-*dysc*/Gal4 driver combinations lead to large, significant reductions in light-phase sleep time (Figure 5D,E,H), each associated with significantly reduced sleep bout times (Supplementary Table S12). Strong effects on waking activity and bout number in the light period were also identified, but each was seen in just one UAS-*dysc*/Gal4 driver combination and may represent genotype-specific effects. One of the *dysc* pan-neuronal knockdowns shows a modest increase in dark-phase sleep time (blue curve, Figure 5D) which is not replicated by any other *dysc* RNAi, potentially suggesting that it is a chance effect (the *p*-value does not survive an experimentwise Bonferroni correction for multiple tests). Indeed, one of the other genotypes shows a dramatic decrease in dark-phase sleep time (Figure 5E) associated with more, but shorter bouts of sleep (Supplementary Table S12). Given that QG10, which harbors *dysc*, encompasses QTL for a number of traits (Table 1), RNAi effects on several sleep traits are not unexpected. However, radical knockdown of expression via RNAi may have led to this generalized impact on several traits, not strictly replicating the effects of any naturally-segregating pleiotropic allele at *dysc*, or the action of multiple segregating *dysc* alleles, each conferring trait-specific effects.

## 4. Conclusions

Using genotypes derived from the DSPR multiparental population, we used a high-throughput behavioral phenotyping approach to identify a number of regions of the *Drosophila* genome that are significantly associated with heritable sleep and activity-related phenotypes. A limitation of our study is our reliance on phenotypes derived from an activity-based monitoring system, where the movement of a fly is assessed by the number of times it breaks a single infrared beam that passes through the middle of a narrow assay tube (5 mm diameter × 65 mm length). This form of behavioral monitoring is very common in the *Drosophila* literature and is extremely convenient for a large screen. Nonetheless, work pairing beam break data with video recordings shows that the former can overestimate sleep time, since flies can be moving and awake, but fail to cross the beam [58]. A related result has been observed when comparing a system with a single infrared beam to one using many beams spanning the length of the assay tube: flies appear to sleep less when there are more beams [59]. Additionally, flies have been shown to have an uneven pattern of activity along the length of activity monitoring assay tubes [60], with some evidence of strain-to-strain variation in a preference to sleep nearer the end of the tube containing media [13,60,61]. As executed, our study would not have been able to discriminate such nuanced behaviors.

However, despite the potential for our study to have missed some important aspects of *Drosophila* sleep and activity, we were able to point to numerous genes with genetic and/or transcriptional variation associated with the traits we did measure. We deliberately focused on genes within mapped sleep QTL that have already been functionally implicated in controlling sleep and/or circadian rhythm and validated the role of some of these genes in sleep via RNAi. But many other genes are present within mapped QTL and possess *cis*-eQTL in head tissue or show a correlation between transcription level in the head and one of our measured sleep phenotypes. The number and complexity of the effects imply that many genes and many pathways contribute to variation in sleep. This view is supported by the observation that our study reveals a largely distinct genetic architecture from a previous sleep genomewide mapping study employing the DGRP lines [6]. Similarly, an artificial selection study initiated from DGRP strains with extreme sleep phenotypes observed little overlap at the polymorphism or gene level between loci identified in the GWAS and loci showing allele frequency changes following selection [22]. Nonetheless, we leveraged our mapping data, together with extant expression data, to point to three strong candidate genes, and our data suggest there is a link between natural variation in these genes and sleep phenotypes in flies.

## Figures and Tables

**Table 1:** Details of mapped QTL groups.

QTL Group	Phenotypes *^a^*	Chr	Interval Size (Mb) *^b^*	Num Genes *^c^*	Num Local eQTL *^d^*
QG1	DBN, DBL	X	0.70	57	26
QG2	DST	2L	1.91	240	74
QG3	DBN	2L	0.60	66	18
QG4	LBN	2L	2.03	158	50
QG5	LWA, LBN	2L	1.17	74	16
QG6	LST	2L	0.75	103	52
QG7	DST, DBN, LBL, DBL	2c *^e^*	7.92	316 *^e^*	178 *^e^*
QG8	DBL	3L	0.90	85	30
QG9	LBL	3L	0.27	35	17
QG10	LST, DST, LBN, DBL	3L	0.64	48	23
QG11	LWA, DWA, LBN	3R	1.21	124	41
QG12	DWA	3R	0.85	84	27

*^a.^* The phenotypes generating a QTL that overlaps with a QTL group (DST and LST = dark-phase and light-phase sleep time; DWA and LWA = dark and light waking activity; DBN and LBN = dark and light bout number; DBL and LBL = dark and light bout length). *^b.^* The size is given based on release 6 of the *Drosophila* *melanogaster* reference genome. For those QTL groups composed of multiple QTL, the interval presented is the minimum interval defined by all overlapping QTL. See Supplementary Table S4 for trait-specific QTL information. *^c.^* The number of protein-coding genes implicated by the QTL group interval. *^d.^* The number of genes within the QTL group interval that have a local (or *cis*) eQTL identified by King et al. [25]. *^e.^* This QTL overlaps the chromosome 2 centromere. In total, 119/316 of the implicated genes encode histone protein subunits, and 111/178 genes with local eQTL are histone genes.

## Supplementary Materials

The following are available online at https://www.mdpi.com/2073-4425/11/3/294/s1, Figure S1: Sleep time assay is robust to batch-to-batch variation, Figure S2: Sleep time distribution among DSPRF1 heterozygous genotypes and among DGRP inbred lines, Figure S3: Sleep time phenotypes from a set of heterozygous genotypes compared to the inbred strains from which they were derived, Figure S4: Correlation between dark and light sleep time genotype means for the full DSPRF1 dataset (N = 787 genotypes), Figure S5: Founder strain effects at trait-specific mapped QTL, Table S1: DSPRF1 genotype means for all measured phenotypes, Table S2: Correlations among traits measured on 787 DSPRF1 genotypes, Table S3: Raw LOD score data for all eight phenotypes mapped in the DSPRF1 population, Table S4: Details on all QTL mapped for sleep and activity traits in the DSPRF1 panel, Table S5: Permutation-derived critical thresholds for QTL mapping of each sleep/activity trait, Table S6: Founder strain effects estimated at all 22 trait-specific QTL, Table S7: Protein-coding genes within the 12 QTL groups, Table S8: FlyBase genes annotated as having a role in sleep and/or circadian rhythms, Table S9: Details of cis-eQTL at genes residing within QTL groups, Table S10: Details of QTL-eQTL founder strain effect correlations, Table S11: Details of the significant correlations between expression level and phenotype, Table S12: Effects of RNAi knockdown on all sleep- and activity-related traits.
