# Supplementary material for: Dissecting the Genetic Basis of Variation in Drosophila Sleep Using a Multiparental QTL Mapping Resource
Source: Genes (Basel). 2020 Mar 11;11(3):294. doi: 10.3390/genes11030294 (PMC7140804; doi:10.3390/genes11030294)
Supplement: Supplementary file 1 [file genes-11-00294-s001.zip › supplementary/Supplementary.docx]

**SUPPLEMENTARY FIGURES AND TABLES**

*For the large data tables only the table legends are presented below; the tables themselves are available for separate download.*

Supplementary Table S1 – tab-delimited text file.

Supplementary Table S2 – Page 3 below.

Supplementary Table S3 – tab-delimited text file.

Supplementary Table S4 – Pages 5-6 below.

Supplementary Table S5 – Page 7 below.

Supplementary Table S6 – tab-delimited text file.

Supplementary Table S7 – tab-delimited text file.

Supplementary Table S8 – tab-delimited text file.

Supplementary Table S9 – tab-delimited text file.

Supplementary Table S10 – tab-delimited text file.

Supplementary Table S11 – tab-delimited text file.

Supplementary Table S12 – Pages 10-11 below.

Supplementary Figure S1 – Page 12 below.

Supplementary Figure S2 – Page 13 below.

Supplementary Figure S3 – Pages 14-15 below.

Supplementary Figure S4 – Page 16 below.

Supplementary Figure S5 – Pages 17-20 below.

**Supplementary Table S1.** DSPRF1 genotype means for all measured phenotypes. Each row gives the mean phenotype associated with a single female genotype, generated by crossing females of the RIL listed in "matRIL" with males of the RIL listed in "patRIL". The "subpop" column indicates the subpopulation from which the genotype is derived, and "N" indicates the number of independent animals tested per genotype. The remaining columns give the mean/SD for each of the measured phenotypes. (Over-batch phenotype averages are given for any genotype scored in replicate batches.) Sleep time and bout time phenotypes are measured in minutes, activity phenotypes are measured in beam-crossings per minute while awake, and bout number is simply an average of counts. Note that the raw bout time phenotypes presented in this table were subjected to a log_10_ transform prior to QTL mapping.

*Table available for separate download.*

**Supplementary Table S2.** Correlations among traits measured on 787 DSPRF1 genotypes.

| **Phenotype *A*** | **Phenotype *B*** | **Correlation *^a^*** | ***P*-value** |
| --- | --- | --- | --- |
| Light sleep time | Dark sleep time | 0.43 | < 10^–36^ |
|  | Light waking activity | –0.23 | < 10^–10^ |
|  | Dark waking activity | 0.07 | 0.06 |
|  | Light bout number | 0.79 | < 10^–40^ |
|  | Dark bout number | –0.32 | < 10^–19^ |
|  | Light bout length | 0.77 | < 10^–40^ |
|  | Dark bout length | 0.38 | < 10^–27^ |
| Dark sleep time | Light waking activity | –0.20 | < 10^–7^ |
|  | Dark waking activity | –0.15 | < 10^–4^ |
|  | Light bout number | 0.36 | < 10^–24^ |
|  | Dark bout number | –0.57 | < 10^–40^ |
|  | Light bout length | 0.33 | < 10^–20^ |
|  | Dark bout length | 0.72 | < 10^–40^ |
| Light waking activity | Dark waking activity | 0.47 | < 10^–40^ |
|  | Light bout number | –0.47 | < 10^–40^ |
|  | Dark bout number | 0.09 | 0.009 |
|  | Light bout length | 0.11 | 0.002 |
|  | Dark bout length | –0.09 | 0.009 |
| Dark waking activity | Light bout number | –0.09 | 0.009 |
|  | Dark bout number | –0.21 | < 10^–8^ |
|  | Light bout length | 0.21 | < 10^–8^ |
|  | Dark bout length | 0.11 | 0.003 |
| Light bout number | Dark bout number | –0.17 | < 10^–5^ |
|  | Light bout length | 0.31 | < 10^–18^ |
|  | Dark bout length | 0.24 | < 10^–10^ |
| Dark bout number | Light bout length | –0.33 | < 10^–21^ |
|  | Dark bout length | –0.84 | < 10^–40^ |
| Light bout length | Dark bout length | 0.34 | < 10^–22^ |

*^a^* Correlation estimated via Pearson's *r*.

**Supplementary Table S3.** Raw LOD score data for all eight phenotypes mapped in the DSPRF1 population. The "Chr" column gives the chromosome for the site under test, "R5" ("R6") is the physical position of the site under test based on Release 5 (6) of the *Drosophila melanogaster* genome, and "cM" is the genetic position. All subsequent "*.LOD" columns give the LOD scores resulting from genetic mapping using the DSPRqtl R package (FlyRILs.org).

*Table available for separate download.*

**Supplementary Table S4.** Details on all QTL mapped for sleep and activity traits in the DSPRF1 panel.

| **Phenotype** | **QTL** | **QTL Group *^a^*** | **LOD** | **Chr** | **Position (cM) *^b^*** | **Interval Size (cM)** | **Interval (Mb) *^c^*** | **Interval Size (Mb) *^c^*** | **Var Expl (%) *^d^*** | **Num Genes *^e^*** |
| --- | --- | --- | --- | --- | --- | --- | --- | --- | --- | --- |
| Light sleep time | Q1 | QG6 | 13.5 | 2L | 53.6 (53.5-53.8) | 0.3 | 19.00-19.75 | 0.75 | 7.6 | 103 |
|  | Q2 | QG10A | 15.5 | 3L | 40.8 (40.1-41.5) | 1.4 | 13.48-14.35 | 0.87 | 8.7 | 58 |
| Dark sleep time | Q1 | QG2 | 10.2 | 2L | 10.3 (6.1-12.4) | 6.3 | 2.54-4.45 | 1.91 | 5.8 | 240 |
|  | Q2 | QG7 | 24.1 | 2R | 54.9 (54.0-55.6) | 1.6 | 20.96(2L)-6.64 *^f^* | 8.34 | 13.2 | 329 *^g^* |
|  | Q3 | QG10B | 10.6 | 3L | 41.3 (39.8-41.7) | 1.9 | 13.28-14.46 | 1.18 | 6.0 | 96 |
| Light waking activity | Q1 | QG5 | 11.5 | 2L | 52.6 (52.3-53.1) | 0.8 | 16.92-18.24 | 1.32 | 6.5 | 89 |
|  | Q2 | QG11C | 16.2 | 3R | 47.7 (47.0-48.8) | 1.9 | 4.26-9.41 | 5.15 | 9.1 | 605 |
| Dark waking activity | Q1 | QG11B | 11.6 | 3R | 47.2 (47.0-47.2) | 0.2 | 4.26-5.47 | 1.21 | 6.6 | 124 |
|  | Q2 | QG12 | 11.9 | 3R | 65.8 (65.0-67.3) | 2.3 | 19.16-20.01 | 0.85 | 6.7 | 84 |
| Light bout number | Q1 | QG4 | 10.5 | 2L | 51.1 (49.5-51.8) | 2.3 | 14.21-16.24 | 2.03 | 6.0 | 158 |
|  | Q2 | QG5 | 11.0 | 2L | 52.7 (52.1-53.0) | 0.9 | 16.73-18.09 | 1.36 | 6.3 | 112 |
|  | Q3 | QG10A | 15.7 | 3L | 40.8 (40.1-41.2) | 1.0 | 13.48-14.12 | 0.64 | 8.8 | 48 |
|  | Q4 | QG11A | 12.2 | 3R | 47.0 (47.0-47.2) | 0.2 | 4.26-5.48 | 1.22 | 6.9 | 126 |
| Dark bout number | Q1 | QG1 | 9.8 | X | 46.3 (44.6-47.6) | 3.1 | 13.72-14.50 | 0.78 | 5.6 | 72 |
|  | Q2 | QG3 | 9.7 | 2L | 36.3 (35.0-37.9) | 2.8 | 9.20-9.80 | 0.60 | 5.5 | 66 |
|  | Q3 | QG7 | 17.4 | 2L | 54.0 (53.9-55.8) | 1.9 | 20.41-6.91(2R) *^f^* | 9.16 | 9.7 | 440 *^g^* |
| Light bout length (log_10_) | Q1 | QG7 | 16.3 | 2L | 54.0 (54.0-55.3) | 1.3 | 20.81-6.20(2R) *^f^* | 8.05 | 9.1 | 340 *^g^* |
|  | Q2 | QG9 | 11.4 | 3L | 37.5 (37.2-37.8) | 0.6 | 12.06-12.33 | 0.27 | 6.5 | 35 |
| Dark bout length (log_10_) | Q1 | QG1 | 10.8 | X | 46.3 (44.9-47.6) | 2.7 | 13.79-14.49 | 0.70 | 6.1 | 57 |
|  | Q2 | QG7 | 25.5 | 2L | 54.0 (54.0-55.5) | 1.5 | 20.87-6.59(2R) *^f^* | 8.38 | 13.9 | 330 *^g^* |
|  | Q3 | QG8 | 10.3 | 3L | 4.0 (3.0-6.0) | 3.1 | 2.28-3.18 | 0.90 | 5.9 | 85 |
|  | Q4 | QG10B | 11.2 | 3L | 41.3 (39.8-41.7) | 1.9 | 13.29-14.48 | 1.19 | 6.4 | 95 |

*^a^* Each phenotype-specific QTL interval was examined for overlap with QTL mapped for any other trait. QTL with overlapping 3-LOD drop intervals were assigned to the same numeric group, with numbers assigned based on order along the genome (X, 2L, 2R, 3L, 3R). For instance, light waking activity Q1 and light bout number Q2 overlap, and were assigned to QTL group "QG5". Recognizing that QTL that physically overlap may not represent the same underlying causative factors, we calculated correlation coefficients between the founder strain effects for all pairs of physically overlapping QTL; We anticipate that founder strain effects at QTL for different traits that are caused by the same genetic factor should be correlated. We observed significant correlations at all QTL groups except for (i) QG10 where the four overlapping QTL fall into two pairs with correlated strain effects, "QG10A" and "QG10B", and (ii) QG11, where the three overlapping QTL do not have correlated founder effects, and QTL are distinguished as "QG11A", "QG11B", and "QG11C".

*^b^* The position of the QTL peak is given, as is the 3-LOD drop interval (in parentheses).

*^c^* The 3-LOD drop interval and the size of the implicated interval is given relative to Release 6 of the *D. melanogaster* reference strain.

*^d^* The percentage of broad-sense heritability of genotype means explained by the QTL.

*^e^* The number of protein-coding genes residing within each mapped QTL interval.

*^f^* These QTL span the Chromosome 2 centromere.

*^g^* These QTL intervals include 119 genes that encode histone proteins.

**Supplementary Table S5.** Permutation-derived critical thresholds for QTL mapping of each sleep/activity trait.

|  | **QTL LOD Threshold** | | | |
| --- | --- | --- | --- | --- |
| **Phenotype** | **1%** | **5% *^a^*** | **10%** | **20%** |
| Light sleep time | 12.4 | 10.7 | 10.2 | 9.5 |
| Dark sleep time | 11.6 | 10.1 | 9.5 | 9.0 |
| Light waking activity | 11.5 | 10.8 | 10.1 | 9.5 |
| Dark waking activity | 11.0 | 9.9 | 9.5 | 8.9 |
| Light bout number | 10.7 | 9.8 | 9.2 | 8.8 |
| Dark bout number | 10.9 | 9.6 | 9.1 | 8.6 |
| Light bout length (log_10_) *^b^* | 11.5 | 10.3 | 9.7 | 9.2 |
| Dark bout length (log_10_) *^b^* | 11.5 | 10.0 | 9.4 | 9.0 |

*^a^* 5% thresholds were used to define significant QTL throughout this work. The other values are provided for context when examining LOD profiles.

*^b^* LOD profiles obtained when using both the raw phenotypic values and the log_10_-transformed values for both bout time traits are similar. However, the permutation derived thresholds are inflated using the raw values, likely due to the heteroscedasticity in the distributions; The 5% threshold for raw light bout length is 19.2, while that for raw dark bout length is 15.0.

**Supplementary Table S6.** Founder strain effects estimated at all 22 trait-specific QTL. The "phenotype" column provides the name of the trait mapped, and the "QTL" and "QTLgroup" columns provide the ID number of the QTL (from left to right along the genome), and the group to which the QTL belongs, respectively. The "N" columns give the number of genotypes harboring each of the founder haplotypes, the "Mean" columns give the phenotype mean for each founder (the strain effects), and the "SE" columns provide the standard error on each mean. For plots and correlations, only means associated with sample sizes >9 were employed.

*Table available for separate download.*

**Supplementary Table S7.** Protein-coding genes within the 12 QTL groups. The "QTLgroup" column gives the identifier for each QTL group (see in-text Table 1). The cytological and physical positions (in *D. melanogaster* reference release 6 coordinates) for each gene implicated by a QTL group interval are given in "Gene.CytLoc" and "Gene.PhysLoc", respectively. The gene symbol and FlyBase identifier are given in the columns "Gene.Symbol" and "FBgn", respectively. Data from FlyBase was accessed in July 2016. The "sleepGene", "cis.eQTL", "ExpCor", and "HarbisonGWAS" columns are 0/1 vectors indicating whether the gene is a recognized sleep/circadian rhythm gene in FlyBase (see Supplementary Table S8), harbors a *cis*-eQTL (from King et al. 2014, PMID: 24810915), shows a significant correlation between its expression level in heads (also from King et al. 2014, PMID: 24810915) and phenotypic variation, or harbors an association with phenotype in the DGRP (from Harbison et al. 2013, PMID: 23617951), respectively.

*Table available for separate download.*

**Supplementary Table S8.** FlyBase genes annotated as having a role in sleep and/or circadian rhythms. The columns "FBgn", "CGnum", "GeneName", "GeneSymbol", all provide information about the gene. "CytLoc" provides the cytological location of the gene. "Chr", "MinPhysLoc_R6", and "MaxPhysLoc_R6" give the physical location of the gene (in *D. melanogaster* reference release 6 coordinates). "WithinQTLgroup" states if the gene is present within a QTL group (giving the name of the group) or not. The FlyBase search terms used to compile this list are provided in the in-text "Results" section. Data from FlyBase was accessed in July 2016.

*Table available for separate download.*

**Supplementary Table S9.** Details of *cis*-eQTL at genes residing within QTL groups. "QTLgroup" identifies the QTL group (see in-text Table 1). "GeneCytLoc" and "GeneR6interval" give the cytological and physical (in *D. melanogaster* release 6 coordinates) positions of the target gene, respectively. "Symbol" and "FBgn" provide the formal identifiers of the gene, while "InternalTranscriptID" gives a lab code for each of the transcripts interrogated. "eQTL.LOD" gives the LOD score of the mapped *cis*-eQTL. "eQTL.Chr" gives the chromosome arm on which the eQTL resides. The physical and genetic (in cM) positions of the *cis*-eQTL peaks are given in the "eQTL.PhysPeak" and "eQTL.cMPeak" columns, and the limits of the 3-LOD drop intervals provided in the "eQTL.PhysIntMin", "eQTL.PhysIntMax", "eQTL.cMIntMin", and "eQTL.cMIntMax" columns. The "N" columns give the number of genotypes harboring each of the founder haplotypes, the "Mean" columns give phenotype mean for each founder (the strain effects), and the "SE" columns provide the standard error on each mean. Note that *cis*-eQTL mapped for different transcripts belonging to the same gene each have their own row in the table. All eQTL data comes from King et al. 2014 (PMID: 24810915).

*Table available for separate download.*

**Supplementary Table S10.** Details of QTL-eQTL founder strain effect correlations. "QTLgroup" refers to the QTL group (see in-text Table 1), "Pheno" to the specific phenotype yielding a QTL overlapping the current QTL group, and "QTLpheno" provides an identifier for the QTL mapped for the current trait. The columns "eQTL.ID", "eQTL.Symbol", and "eQTL.FBgn", provide information on the transcript/gene yielding the *cis*-eQTL. Since eQTL for a given gene can be identified for multiple transcript isoforms, a given gene can be represented in the file multiple times, associated with different IDs. "Pearson.r" provides the correlation coefficient between the eQTL founder strain effects and the phenotype-specific QTL strain effects, "Nfounder" provides the number of founders possessing strain effects for both QTL and eQTL, "Pval" provides the *p*-value of the correlation. Only strain effects resulting from at least 10 RILs are used in the correlations.

*Table available for separate download.*

**Supplementary Table S11.** Details of the significant correlations between expression level and phenotype. Expression levels are from fro King et al. 2014 (PMID: 24810915). Only correlations significant at a per-trait 5% FDR level are presented. "Pheno" indicates which phenotype the correlation is for, noting that for light and dark bout time the raw - rather than the log_10_ transformed - values were employed. "FBgn" and "Symbol" give details on the gene, and "ID" gives the identifier for the transcript. "Pearson.r" provides the correlation coefficient between the phenotype score and the expression level, and "Pval" and "Qval" give the significance values. The final column "GeneUnderQTL" states which - if any - QTL group the gene is associated with.

*Table available for separate download.*

**Supplementary Table S12.** Effects of RNAi knockdown on all sleep- and activity-related traits.

| **UAS Code** *^a^* | **UAS Stock** *^b^* | ***elav* Driver** *^c^* | **N** *^d^* | **Test** *^e^* | **Light Sleep Time** *^f^* | **Dark Sleep Time** *^f^* | **Light Waking Activity** *^g^* | **Dark Waking Activity** *^g^* | **Light Bout Number** | **Dark Bout Number** | **Light Bout Length** *^h^* | **Dark Bout Length** *^h^* |
| --- | --- | --- | --- | --- | --- | --- | --- | --- | --- | --- | --- | --- |
| tim (1) | TRiP 29583 | Gal4 | 127 / 62 | Diff | −121.3 | −32.7 | 0.29 | 0.17 | −3.04 | 0.75 | −0.18 | −0.06 |
|  |  |  |  | *P* | < 10^−14^ *** | 0.01 * | < 10^−5^ *** | < 10^−3^ *** | < 10^−3^ *** | 0.14 | < 10^−9^ *** | 0.04 * |
|  |  | GS-Gal4 | 128 / 64 | Diff | −27.6 | −28.9 | 0.01 | −0.03 | −2.80 | 2.12 | 0.04 | −0.17 |
|  |  |  |  | *P* | 0.02 * | < 10^−3^ ** | 0.73 | 0.43 | < 10^−6^ *** | < 10^−4^ *** | 0.09 | < 10^−6^ *** |
| tim (2) | TRiP 40864 | Gal4 | 127 / 76 | Diff | −134.5 | 9.4 | −0.11 | 0.08 | −2.63 | 0.23 | −0.27 | 0.00 |
|  |  |  |  | *P* | < 10^−17^ *** | 0.37 | < 0.01 ** | 0.03 * | < 10^−3^ ** | 0.63 | < 10^−17^ *** | 0.96 |
|  |  | GS-Gal4 | 128 / 128 | Diff | −73.3 | −6.7 | 0.00 | 0.05 | −2.74 | 1.74 | −0.03 | −0.13 |
|  |  |  |  | *P* | < 10^−8^ *** | 0.36 | 0.93 | 0.16 | < 10^−7^ *** | < 10^−4^ *** | 0.19 | < 10^−4^ *** |
| Ddc (1) | KK-VDRC 109881 | Gal4 | 147 / 86 | Diff | 64.0 | −5.1 | −0.29 | −0.01 | 2.17 | −0.05 | 0.07 | −0.01 |
|  |  |  |  | *P* | < 10^−5^ *** | 0.67 | < 10^−9^ *** | 0.84 | < 10^−3^ *** | 0.90 | 0.01 * | 0.85 |
|  |  | GS-Gal4 | 128 / 127 | Diff | 42.3 | 4.0 | −0.21 | 0.05 | 2.34 | 0.88 | −0.01 | −0.05 |
|  |  |  |  | *P* | < 10^−3^ *** | 0.56 | < 10^−9^ *** | 0.11 | < 10^−7^ *** | 0.02 * | 0.58 | 0.14 |
| Ddc (2) | TRiP 27030 | Gal4 | 127 / 64 | Diff | −3.8 | −1.4 | 0.10 | 0.09 | 0.81 | −0.02 | −0.02 | 0.00 |
|  |  |  |  | *P* | 0.82 | 0.90 | 0.04 * | 0.02 * | 0.30 | 0.98 | 0.54 | 1.00 |
| Ddc (3) | TRiP 51462 | Gal4 | 127 / 64 | Diff | 70.9 | 46.2 | −0.16 | 0.06 | 5.18 | −2.76 | 0.00 | 0.17 |
|  |  |  |  | *P* | < 10^−4^ *** | < 10^−5^ *** | < 0.01 ** | 0.08 | < 10^−8^ *** | < 10^−8^ *** | 0.92 | < 10^−7^ *** |
| dysc (1) | GD-VDRC 14082 | Gal4 | 124 / 61 | Diff | −50.9 | −13.9 | −0.09 | −0.02 | −0.5 | 1.52 | −0.14 | −0.07 |
|  |  |  |  | *P* | < 10^−4^ *** | 0.25 | 0.04 * | 0.54 | 0.36 | < 0.01 ** | < 10^−5^ *** | 0.03 * |
|  |  | GS-Gal4 | 126 / 64 | Diff | −54.2 | −15.7 | −0.14 | −0.01 | 1.21 | 2.98 | −0.12 | −0.18 |
|  |  |  |  | *P* | < 10^−6^ *** | 0.07 | < 10^−4^ *** | 0.67 | 0.02 * | < 10^−7^ *** | < 10^−4^ *** | < 10^−6^ *** |
| dysc (2) | GD-VDRC 28945 | Gal4 | 124 / 63 | Diff | −39.2 | 37.8 | −0.01 | 0.10 | −0.45 | −0.21 | −0.10 | 0.05 |
|  |  |  |  | *P* | < 0.01 ** | < 0.01 ** | 0.79 | 0.04 * | 0.40 | 0.68 | < 0.01 ** | 0.11 |
| dysc (3) | KK-VDRC 11019 | Gal4 | 147 / 64 | Diff | −100.7 | −110.2 | 0.08 | 0.00 | −4.98 | 2.52 | −0.18 | −0.24 |
|  |  |  |  | *P* | < 10^−14^ *** | < 10^−11^ *** | 0.09 | 0.95 | < 10^−13^ *** | < 10^−8^ *** | < 10^−7^ *** | < 10^−12^ *** |

Effects of RNAi knockdown of three candidate genes, *timeless* (tim), *Dopa decarboxylase* (Ddc), and *dychronic* (dysc). The difference ("Diff") in mean phenotype between the RNAi knockdown genotype and its respective control is provided, along with the *P*-value from the associated *t*-test comparing the two. Red indicates evidence that trait value is reduced following knockdown, and green indicates evidence trait value is increased following knockdown.

*^a^* Codes match those also used in in-text Figure 5.

*^b^* The BDSC (TRiP strains) or VDRC (GD- and KK-VDRC strains) stock number providing the UAS-RNAi transgene. Experimental TRiP genotypes were compared to control BDSC stock number 35788, experimental KK-VDRC genotypes were compared to control VDRC stock number 60100, and experimental GD-VDRC genotypes were compared to control VDRC stock number 60000.

*^c^* The *elav* driver employed; "Gal4" is a standard *elav*-Gal4 driver, while "GS-Gal4" is an RU486-inducible GeneSwitch driver.

*^d^* Number of individuals assayed for each genotype. The value before the slash is the number of control individuals, the value after the slash is the number of experimental individuals.

*^e^* "Diff" values give the difference between the averages of the experimental genotype and the appropriate control. Negative values imply a reduction in trait value following RNAi knockdown, while positive values imply an increase in trait value on knockdown. "P" provides the *P*-values from Welch's *t*-tests comparing control to experimental genotypes (*, *P* < 0.05; **, *P* < 0.05/12 - a trait-specific Bonferroni threshold; ***, *P* < 0.05/96 - an experimentwise Bonferroni threshold.) "Diff" values significant at either a trait-specific or experimentwise threshold are colored by the direction of the change.

*^f^* Sleep time is measured in minutes.

*^g^* Activity level, measured as the number of infrared beam crosses, per minute while awake.

*^h^* As for the QTL analysis, both bout time phenotypes were subjected to a log_10_ transform prior to analysis.

**Supplementary Figure S1.** Sleep time assay is robust to batch-to-batch variation. Fifty-one DSPRF1 genotypes were assayed over 2 experimental batches. The figure shows mean sleep time (+/– 1SD) for each replicated genotype in both the light and dark period, calculated separately for each batch. Correlation among batches is evident in the similar average phenotype generated by each genotype in each batch (light sleep time, *r* = 0.72, *p* < 10^–8^; dark sleep time, *r* = 0.70, *p* < 10^–7^). For the purposes of this figure genotypes are arbitrarily assigned numeric codes, and the genotypes are ordered by phenotype independently for each plot.

**Supplementary Figure S2.** Sleep time distribution among DSPRF1 heterozygous genotypes and among DGRP inbred lines. Histograms of mean female sleep time for all 787 DSPRF1 genotypes and 168 DGRP lines (latter data taken directly from Harbison et al. 2013, PMID: 23617951, Additional File 3). Average sleep time over genotypes for each dataset is presented above each histogram (solid red circle) along with 1-SD values (red lines).

**Supplementary Figure S3.** Sleep time phenotypes from a set of heterozygous genotypes compared to the inbred strains from which they were derived. The plots on this page show the mean sleep time for 30 DSPRF1 genotypes (as ×), and the mean sleep time for the associated pair of parental RILs (female inbred parent, pink; male inbred parent, blue). A gray bar is shown between the parental RIL phenotypes to more easily visualize the relationship between the phenotype of each outbred genotype to the associated inbred genotypes. Phenotypes of all genotypes depicted were generated in the same way as for the DSPRF1 mapping panel.

**Supplementary Figure S3 (contd).** The plots on this page show the mean sleep time for 22 F_1_ genotypes (as ×), and the mean sleep time for the associated pair of parental DGRP inbred lines (female inbred parent, pink; male inbred parent, blue). Phenotypes of all genotypes depicted were generated in the same way as for the DSPRF1 mapping panel.

**Supplementary Figure S4.** Correlation between dark and light sleep time genotype means for the full DSPRF1 dataset (N=787 genotypes). The correlation is highly significant (Pearson's *r* = 0.43, *p* < 10^–40^).

**Supplementary Figure S5.** Founder strain effects at trait-specific mapped QTL. Data is only presented for founders present in at least 10 RILs at a probability > 0.95. AB8 was used to found both synthetic populations, so is depicted twice in each figure. Units for each phenotype are as follows: light/dark sleep time (minutes), light/dark waking activity (beam crosses per minute), light/dark bout number (count), light/dark bout length (minutes, following a log_10_ transform). For each QTL we give both the phenotype-specific QTL code, and the QTL group (see Supplementary Table S4).
